# Gastro-Protective and Anti-Oxidant Potential of *Althaea officinalis* and *Solanum nigrum* on Pyloric Ligation/Indomethacin-Induced Ulceration in Rats

**DOI:** 10.3390/antiox8110512

**Published:** 2019-10-25

**Authors:** Sameh S. Zaghlool, Ali A. Abo-Seif, Mohamed A. Rabeh, Usama Ramadan Abdelmohsen, Basim A. S. Messiha

**Affiliations:** 1 Pharmacology and Toxicology Department, Faculty of Pharmacy, Modern University for Technology and Information (MTI), Mokattam, Cairo 11571, Egypt; 2 Pharmacology and Toxicology Department, Faculty of Pharmacy, Nahda University (NUB), Beni-Suef 62511, Egypt; ali.aboseif@nub.edu.eg; 3 Department of Pharmacognosy, Faculty of Pharmacy, Cairo University, Cairo 11562, Egypt; mohamed.rabeh@pharma.cu.edu.eg; 4 Department of Pharmacognosy, Faculty of Pharmacy, Deraya University, Universities Zone, New Minia City 61111, Egypt; 5 Department of Pharmacognosy, Faculty of Pharmacy, Minia University, Minia 61519, Egypt; 6 Pharmacology and Toxicology Department, Faculty of Pharmacy, Beni-Suef University, Beni-Suef 62514, Egypt; drbasimanwar2006@yahoo.com

**Keywords:** *Solanum nigrum*, *Althaea officinalis*, gastric ulcer, indomethacin, anti-oxidant activity

## Abstract

Recently, an alternative disease treatment approach is the research of medicaments from traditional medicine. Plants with anti-oxidant capabilities are used as herbal treatments for ulcer diseases. Medicinal/herbal extracts containing phytoconstituents have significant anti-ulcer activities in in vivo experiments on animal models, compared to reference drugs. The current study aims to inspect gastro-protective as well as in vitro and in vivo anti-oxidant potential of *Althaea officinalis* and *Solanum nigrum* extracts on pyloric-ligation/indomethacin-induced gastric-ulceration in rats. Rats were divided into six groups: normal control, gastric ulcer control, two standard pretreatment groups receiving omeprazole and misoprostol, and two test pretreatment groups receiving *Althaea officinalis* and *Solanum nigrum*. Pretreatments were administrated orally for 14 days. On the 15th day, animals, excluding the normal control group, were exposed to pyloric-ligation followed by indomethacin injection. After four hours, the rat’s stomachs were removed and gastric juice and blood samples were collected. Pyloric-ligation/indomethacin administration caused considerable elevation in ulcer number, ulcer index, acid and pepsin productivity, aggressive factors, and gastric mucosal lipid-peroxide contents. Moreover, reduction in titratable acidity, gastric mucosal nitric-oxide, anti-oxidant contents, and protective factors accompanied gastric-ulceration. Additionally, elevation in pro-inflammatory cytokines content and reduction in cystathionine-β-synthase and heme-oxygenase-1 expression was witnessed. Omeprazole, misoprostol, *Althaea officinalis,* and *Solanum nigrum* pretreatments fixed blood and tissue biomarkers, thereby protecting them from pyloric-ligation/indomethacin-induced gastric-ulceration in rats, which is hopeful for clinical examinations.

## 1. Introduction

Today, gastric hyperacidity and gastroduodenal ulcers represent a serious global problem [1]. A gastric ulcer is generated by an imbalance between the defense system and a damaging force in the gastroduodenal mucosa. Damaged mucosal defense is found in gastric ulcer patients with normal gastric acid and pepsin contents. Patients regularly using non-steroidal anti-inflammatory drugs (NSAIDs) must be advised about mucosal prostaglandin synthesis suppression and consequently increased ulcer risk [2].

Oxidative stress and free radical-mediated processes have been involved in the pathogenesis of gastrointestinal disturbances [3]. NSAID’s, including indomethacin, are known as the major causative factors of gastric ulcers [4,5,6]. The generation of the gastric ulceration induced by indomethacin takes place by inhibition of protective factors like prostaglandin-E_2_ (PG-E_2_), bicarbonate, and mucus, together with potentiation of aggressive factors including increased gastric hyperacidity and oxidative stress [6,7,8].

Proton pump inhibitors (PPIs), including omeprazole, are frequently used for therapeutic management of acid-related disorders such as gastroesophageal reflux disease and Zollinger–Ellison syndrome and for the management of peptic-ulcer diseases induced by stress, NSAIDs, and *Helicobacter pylori* infection. Inhibiting the secretion of gastric acid by PPIs is the major way to manage these disorders. PPIs inhibit gastric acid secretion via irreversible interaction with the proton pump (H^+^/K^+^-ATPase) of the parietal cell, offering potent anti-ulcer activity. Still, whether PPIs have a role in regulating damage by inhibition of oxidative stress or by inhibition of gastric acidity only is not well known [9,10]. Omeprazole recovers gastric and duodenal ulcers highly effectively [10].

Misoprostol, a prostaglandin-E_1_ analogue, can promote gastric ulcer healing in the presence of NSAIDs through several mechanisms. These include suppressing acid secretion, elevating prostaglandin contents in the stomach, and stimulating or modulating factors implicated in the healing of ulcers such as angiogenesis, epithelial cell regeneration, wound contraction, and blood flow [11].

Since long ago, natural products from traditional medicine have represented an inspiration for the development of new drugs [12]. The use of herbal drugs for the treatment of gastrointestinal disorders, as a part of complementary and alternative medicine (CAM), is increasing in developed and developing countries. The World Health Organization (WHO) recommends medicinal plants for which scientific evidence is established for their safety and therapeutic efficacy [13]. Medicinal plants and their extracts have an important vital role against many diseases. Herbs represent excellent resources for cost-effective and readily available gastro-protective remedies without significant side effects [14,15,16,17]. Medicinal/herbal plants and extracts represent some of the most attractive resources of new drugs and have shown promising results for the treatment of gastric ulcers [17,18,19,20,21,22,23,24]. The natural products obtained from medicinal plants, including flavonoids, polyphenols, terpenoids [18], saponins [24], alkaloids [12], and mucilaginous polysaccharides [25], show immense pharmacological significance such as anti-inflammatory, antimicrobial, antiulcer, anti-oxidant, and anticancer activities [24,26,27]. In in vivo experiments on animal models showed that many plants and their extracts have considerable anti-ulcerogenic properties [16] through anti-oxidant, muco-protective, and gastric anti-secretory activity in comparison with that of reference drugs [28]. Plant extracts are generally safe even at high concentrations. The anti-ulcer properties of plants may be attributed to the flavonoid [14,28,29] and triterpene contents [30].

Anti-oxidants can scavenge free radicals before attacking cells and biological targets [31]. Therefore, their activity will be critical for maintaining optimal protection. Anti-oxidant products can be synthetic or natural [32]. Therefore, a considerable interest is critical to find natural anti-oxidants from herbal origins to replace synthetic ones for maintaining optimal protection against many disorders, such as gastric ulcers [18,33].

Polyphenols (mainly tannins and flavonoids) have been associated with anti-inflammatory, anti-oxidant, and immunomodulatory properties. Through reducing oxidative stress, polyphenols can alter gene expression related to inflammation, suppressing downstream cytokine formation (e.g., TNF-α, and IL-1β), and upgrading the tissue anti-oxidant enzymes superoxide-dismutase (SOD) and reduced glutathione (GSH) [26]. Phenolic compounds were reported to exhibit anti-ulcerogenic activities. They act through different mechanisms, such as anti-secretory effects or cytoprotective effects, as they increase PG synthesis [24]. Polyphenols are considered natural anti-oxidant compounds as they have serious potential for protection against tissue harm induced by free radicals [27]. Flavonoids can decrease histamine secretion from mast cells and inhibit lipid peroxidation as well [18,24]. Additionally, flavonoids maintain the gastric mucosal glycoprotein moiety, and may cause an increase in nitric oxide (NO) action [18]. Tannins have astringent activities, precipitating proteins of mucosal membranes and skin. Some types of tannins suppress gastric secretion and enhance the mucus layer, and have a local action of protection of the gastric mucosa [7,26]. 

Polysaccharides are cytoprotective agents that stimulate mucosal regeneration and proliferation and increase PG synthesis, restoring the gastric mucus levels [24].

Saponins were reported to possess anti-ulcer activity in several experimental models, possibly through activation of mucous membrane protective factors [18]. Saponins act mainly through anti-secretory mechanisms as they inhibit acid secretion and total acid output and lower the pH value of gastric juice [24].

Triterpenoids have several pharmacological activities, including anti-inflammatory and anti-ulcer activities [18]. Terpenoids with anti-ulcerogenic effects are mostly cytoprotective. They increase mucus production and PG content, and improve gastric mucosal blood flow and bicarbonate secretion. Additionally, they accelerate ulcer healing by anti-oxidant activity through reduction of lipid peroxide content and elevation of SOD activity in the stomach [24].

Alkaloids have a gastro-protective activity, which may involve the participation of sulfhydryl compounds, NO and PG, as well as reduction of IL-1β and TNF-α contents and elevation of GSH contents [12]. Most of the reported alkaloids possess prophylactic anti-ulcerogenic activity through enhancing mucus production in addition to the anti-oxidant activity [24].

*Althaea officinalis* L. (Marshmallow), a member of Malvaceae family, is well-known for its medicinal properties. The aqueous extract of *Althaea officinalis* was demonstrated to be potentially helpful in treating lipemia, inflammation, gastric ulcers, and platelet aggregation with no detected adverse or toxic effects [25]. The pharmacological and anti-oxidant activities of *Althaea officinalis* were attributed to various compounds such as polysaccharides and flavonoids present in the plant [34,35].

*Solanum nigrum* L., family: *Solanaceae* (Black nightshade) has been extensively used in traditional medicine. Previous investigations documented that *Solanum nigrum* fruits have many valuable activities like anti-ulcer and anti-oxidant activities, as well as suppression of lipid peroxidation. *Solanum nigrum* extract, when administrated orally, yielded a considerable anti-ulcer activity with no visible toxicological effects. Additionally, aerial parts of *Solanum nigrum* can reduce gastric acid secretion, diminish pepsin activity, and elevate mucus release [1,5].

The current investigation aims to determine the gastro-protective and anti-oxidant potential of extracts of *Althaea officinalis* and *Solanum nigrum* on pyloric-ligation/indomethacin-induced gastric-ulceration in rats. 

## 2. Materials and Methods 

### 2.1. Animals

Adult male albino rats (200–250 g) were used in the present investigation. Animals were obtained from the animal house of Nahda University, Beni-Suef, Egypt. Animals were kept under observation for about 15 days before starting the experimental protocol to exclude any inter-current infection. The chosen animals were housed in plastic cages with good aerated covers in a controlled environment, at a constant temperature (25 ± 0.5 °C) and light/dark (12/12 h) cycles. Animals were allowed free access to water and standard forage ad libitum. All animal housing and handling were conducted in compliance with the Nahda University (NUB) guidelines and in accordance with the research protocols recognized by the Animal Care Committee of the National Research Center (Cairo, Egypt) which comply with the recommendations of the National Institutes of Health (NIH) guide for care and use of laboratory animals and were approved by the Ethics Committee (NUB#002-019 on 07/05/2019).

### 2.2. Plant Materials

*Althaea officinalis* flowers were obtained from the Haraz Company (Cairo, Egypt) and *Solanum nigrum* fruits were collected from Beni-Suef farms (Beni-Suef, Egypt) in September 2017. Both of them were authenticated by Dr. Mohamed El-Gibali and Mrs. Therris Labib, senior botanists and head specialists for plant identification at El-Orman Botanical Garden, Giza, Egypt.

Aqueous extracts of *Althaea Officinalis* were prepared by soaking the dried flowers of *Althaea officinalis* (1 kg) in hot water (85–90 °C) for 30 min, followed by filtration and drying of the filtrate under reduced pressure [25], to get a yield of about 11.8% *w*/*w*. A solution of the residue in normal saline was prepared at a concentration of 100 mg/mL, ready for oral administration.

Ethanolic extracts of *Solanum nigrum* were prepared by extraction of the crushed fresh fruits (1 kg) with 96% ethanol, and the extract was evaporated to dryness using a rotary evaporator (IKA^®^ RV 10, Digital, 20–270 rpm, IKA^®^ HB 10, Basic, 0–180 °C, Staufen, Germany), to get a yield of about 7.9% *w*/*w* [36,37]. A solution of the residue in normal saline was prepared at a concentration of 200 mg/mL, ready for oral administration.

### 2.3. Drugs, Chemicals, and Reagent Kits

All used chemicals, solvents, and reagents were purchased and obtained from authorized sources and of analytical grade. Omeprazole was purchased as Omepak^®^ 40 mg capsules (South Egypt Drug Industries Company “SEDICO”, 6th of October City, Egypt). Misoprostol was obtained as a gift from Sigma Pharmaceutical Company, Egypt. Assay: 1.04%. Gastrin (catalog number: MBS704511), histamine (catalog number: MBS732202), SOD (catalog number: MBS036924), PG-E_2_ (catalog number: MBS705028), and PG-I_2_ (catalog number: MBS743424) reagent kits were purchased as the rat ELISA kit from MyBiosource, Inc., Southern California, San Diego, CA, USA. The MDA reagent kit was purchased as the rat ELISA kit (catalog number: LS-F28018) from Life Span BioSciences, Inc., Seattle, Washington, DC, USA. The NO reagent kit (catalog number: 917-010) was purchased as the nitric oxide (NO_2_^−^/NO_3_^−^) assay kit from Assay Designs^TM^, Inc., Ann Arbor City, MI, USA. The GSH reagent kit was purchased as the rat ELISA kit (catalog number: E02G0367) from ShangHai BlueGene Biotech co., LTD., Shanghai, China. The primary antibodies for western blot analysis of TNF-α, IL-1β, CBS, and HO-1 were purchased from Thermo–Fisher Scientific, Rockford, IL, USA. 

### 2.4. Overview of Methodology

To fulfill the purpose of the present investigation, a preliminary phytochemical study of the prepared extracts (aqueous extract of *Althaea officinalis* and ethanolic extract of *Solanum nigrum*) used in this study had to be done followed by evaluation of the in vitro anti-oxidant activity of the extracts (DPPH assay). After the dosing period and collection of tissue, blood, and gastric juice samples, macroscopic examination (ulcer number, ulcer index, and preventive index) and acid productivity assessment (gastric volume, titratable acidity, and acid output) were done. This was accompanied by assessment of aggressive factors like peptic activity, histamine and gastrin contents, and measurement of oxidative stress biomarkers including superoxide-dismutase (SOD) activity and reduced glutathione (GSH), nitric oxide (NO), and malondialdehyde (MDA) contents and protective factors including prostaglandin-E_2_ (PG-E_2_) and prostacyclin (PG-I_2_) contents in accordance with assessment of immunological factors including tumor-necrosis-factor-alpha (TNF-α), interleukin-1-beta (IL-1β), cystathionine-β-synthase (CBS), and heme-oxygenase-1 (HO-1) expressions. All these parameters were assessed via enzyme-linked immunosorbent assay (ELISA) and western blot analysis. Microscopic histopathological examinations were also performed using routine hematoxylin and eosin (H and E) staining and special alcian blue staining for determination of gastric wall mucus (mucin content).

### 2.5. Preliminary Phytochemical Screening of Extracts

The extracts of *Althaea officinalis* and *Solanum nigrum* were subjected to preliminary phytochemical screening [38,39]. These extracts were qualitatively analyzed for the presence or absence of phytochemical constituents employing standard analysis methods. These methods are conducted to detect the presence or absence of carbohydrates by the Molisch test, tannins by the FeCl_3_ test, flavonoids by the NaOH/HCl test, saponins by the froth test, and for glycosides: anthraquinon glycosides by the Born-trager test, cardiac glycosides by the Keller-Killiani test and cyanogenic glycosides by the sodium picrate paper test, sterols and triterpenoids by the Salkowski test, alkaloids by the Dragendorff test, and volatile oils by the Sudan III test and by checking if the extract had a characteristic odor or not.

### 2.6. Evaluation of the In vitro Anti-Oxidant Activity (DPPH Assay) 

The in vitro anti-oxidant potential of test extracts was determined according to the method described by Takao et al. which was modified by Delazar et al. [40,41]. Briefly, a solution of 4 mg DPPH in 50 mL methanol was prepared to get a final concentration of 80 µg/mL. Each extract was diluted in methanol to get different dilutions ranging from 20–1000 µg/mL. A mixture of 1 ml of each diluted extract with DPPH (1 mL) was prepared and stood at room temperature for 30 min and acted as test samples. A mixture of 1 ml of methanol and DPPH (1 mL) was prepared and acted as a control sample. The optical density of all mixtures was determined at 517 nm. Positive control samples using ascorbic acid were prepared by the same method. The DPPH radical scavenging activity of test and positive control samples were calculated according to the following equation:

% Inhibition = [(A_0_ − A_1_)/A_0_] × 100 (1)

where: A_0_ is the optical density of the control sample and A_1_ is the optical density of the test or positive control sample after 30 min. The IC_50_ value was estimated as the concentration (µg/mL) of the test or positive control samples required to scavenge 50% of DPPH.

### 2.7. Experimental Design

Animals were divided randomly into 6 groups, each of 6–8 rats, where standard pretreatments, test pretreatments, or vehicles were administrated orally once daily for 14 days before gastric ulcer induction by the pyloric-ligation/indomethacin administration method. Group I: normal control group, receiving 10 mL/kg normal saline, group II: gastric ulcer control group, receiving 10 mL/kg normal saline together with pyloric-ligation/indomethacin administration, group III: 1st standard pretreatment group, receiving omeprazole (20 mg/kg/day) [7], group IV: 2nd standard pretreatment group, receiving misoprostol (300 µg/kg/day) [11,42], group V: 1st test pretreatment group, receiving *Althaea officinalis* extract (100 mg/kg/day) [25], and group VI: 2nd test pretreatment group, receiving *Solanum nigrum* extract (200 mg/kg/day) [1].

### 2.8. Induction of Gastric Ulcer and Sample Preparation

On the 15th-day of pretreatment or vehicle administration and after 36 hours of starvation, rats were anesthetized with ether inhalation, carefully dissected, and exposed to pyloric-ligation [43] for gastric juice collection. After that, indomethacin (25 mg/kg) [44,45] was injected intra-peritoneally, excluding the normal control group which received a single intra-peritoneal injection of 1% saline solution with Tween 80 (vehicle of indomethacin). After 4 h, rats were killed by cervical dislocation under ether anesthesia. Stomachs from all rats in each group were removed and the gastric juice collected. Blood samples were collected from the retino-orbital sinus. The stomach mucosal tissue was then opened and assessed for macroscopic examination. Acid productivity parameters were determined and gastric mucosal homogenates were prepared in normal saline.

### 2.9. Macroscopic Examination (Assessment of Gross Mucosal Damage)

The glandular part of the stomach was inspected and examined for macroscopical mucosal lesions. The number of lesions was counted using an illuminated magnifying lens (10×) [46,47]. The ulcer index (mm) for each group was determined by the earlier method described by Cho and Olge [48] indicating that the ulcer index is the mean of total ulcer length and hemorrhagic spots in the rats of each group. Hano et al. [49] determined the equation of preventive index calculation as follows:

(2) $$\mathrm{Preventive}\;\mathrm{Index}\;\left(\%\right)=\frac{\mathrm{Ulcer}\;\mathrm{Index}\;\left(\mathrm{Ulcer}\;\mathrm{Control}\;\mathrm{group}\right)-\mathrm{Ulcer}\;\mathrm{Index}\;\left(\mathrm{Treated}\;\mathrm{group}\right)}{\mathrm{Ulcer}\;\mathrm{Index}\;\left(\mathrm{Ulcer}\;\mathrm{Control}\;\mathrm{group}\right)}\times \;100$$

### 2.10. Microscopic Histopathological Examination (Routine H and E Staining and Alcian Blue Staining)

All data and micrographs of microscopic examinations were obtained with the aid of a full HD microscopic camera operated by the Leica application module for tissue section analysis (Leica Biosystems, Wetzlar, Germany).

#### 2.10.1. Routine H and E Staining (Histopathological Examination of Stomach)

The method of routine hematoxylin and eosin (H and E) staining was described by Drury and Wallington for estimating histological assessment of the structure of the stomach tissue [50]. The ulcers, necrotic changes, edema, and inflammatory cell infiltration in the gastric mucosa were determined in all experimental groups.

#### 2.10.2. Alcian Blue Staining for Determination of Gastric Wall Mucus (Mucin Content)

The method of the determination of gastric wall mucus was described by Corne et al. [51] using alcian blue (pH = 2.5) for acidic mucosubstances staining. This method was done with some modifications as it is reported that alcian blue can bind to mucus cells in gastric mucosa, indicating the amount of mucus [52]. The % area of alcian blue positive goblet cells and mucin in gastric mucosa were quantified in 5 random non-overlapping fields per tissue section in a blind manner with total sections (number = 5 per group).

### 2.11. Acid Productivity Parameters (Gastric Volume, Titratable Acidity, and Acid Output Determination)

After centrifugation of the collected gastric juice at 3000 rpm for 10 min, and elimination of solid mass, the volume of gastric juice (mL) was measured. When the volume of solid mass was more than 0.6 mL, the sample was rejected [43]. A portion of 0.2 mL of gastric juice was titrated with 0.01 N sodium hydroxide using phenolphthalein as an indicator to determine titratable acidity as described by Shay et al. [53] and Grossman [54]. After that, acid output was determined according to the method described by Brodie and Hooke [55] as microequivalents per 4 h.

(3) $$\mathrm{Acid}\;\mathrm{Output}\;\left(\mu \mathrm{Eq}/4\mathrm{hr}\right)=\frac{T\;\times \;V}{4}$$

where: (T) is titratable acidity (mEq/L) and (V) is volume of gastric juice collected (mL).

### 2.12. Peptic Activity Determination

Pepsin activity is the major cause of the proteolytic activity of gastric secretion. In brief, this proteolytic activity was measured by the amount of proteases produced after 30 min of incubation of casein substrate with pepsin. This activity of pepsin was measured in gastric juice spectrophotometrically at 280 nm [56].

### 2.13. Biomarkers Estimated Using ELISA Technique

Plasma gastrin as well as gastric mucosal histamine content, oxidative stress biomarkers (gastric mucosal GSH and MDA contents and SOD activity), and protective factors (gastric mucosal PG-E_2_ and PG-I_2_ contents) were estimated using ELISA test reagent kits in compliance with the manufacturer’s instructions at 450 nm.

Assessment of total NO content in gastric mucosal homogenate was estimated using a nitric oxide (NO^2−^/NO^3−^) assay kit in compliance with the method described by Kendrich et al. [57] at 540 ± 20 nm.

These biomarkers were measured by the aid of an ELISA Processing System (model: Spectra Max Plus-384 Absorbance Microplate Reader, San Jose, CA, USA).

### 2.14. Immunological Markers (Gastric Mucosal TNF-α and IL-1β Contents and CBS and HO-1 Activities) Estimated by Western Blot Analysis

Tissue levels of TNF-α, IL-1β, CBS, and HO-1 were measured using the western blot technique described earlier [58,59] via a BioRad mini protein electrophoresis separation unit (model: 1658004, Sinorica international patent and trademark, Germantown, MD, USA). Briefly, a portion of the stomach glandular tissue was subjected to RIPA lysis buffer, and then the lysed samples were brought to complete protein extraction. The lysate was kept in ice for 30 min on a shaker and cell debris was removed by centrifugation at 16,000× *g* using a cooling centrifuge. Supernatants were transferred to new tubes for protein concentration determination. Protein separation by gel electrophoresis was performed using the procedure termed SDS–PAGE (sodium dodecylsulfate–polyacrylamide gel electrophoresis) via the proper antibodies. Briefly, incubation was done overnight in each primary antibody solution, against the blotted target protein, at 4 °C. The blot was rinsed 3–5 times for 5 min with TBST (Tris-buffered saline with Tween 20). Incubation was done in the horseradish peroxidase (HRP)-conjugated secondary antibody solution against the blotted target protein for 1 h at room temperature. The blot was rinsed 3–5 times for 5 min with TBST. The chemiluminescent substrate was applied to the blot according to the manufacturer’s recommendation. The chemiluminescent signals were captured using a CCD camera-based imager. Image analysis software was used to read the band intensity of the target proteins against the normal control sample after normalization by beta-actin on the Chemi-Doc MP Imager.

### 2.15. Statistical Analysis

Statistical analysis and the significance of difference between groups’ means were performed via one-way ANOVA, and after that a Tukey–Kramer multiple comparisons test was done, by the aid of GraphPad Instat computer software (GraphPad Software, San Diego, CA, USA). In addition, Microsoft Excel 2010 software (Microsoft Corporation, Microsoft Redmond campus in Redmond, WA, USA) was used to create graphs and tables.

## 3. Results

### 3.1. Preliminary Phytochemical Screening of Extracts

**Figure 1:**
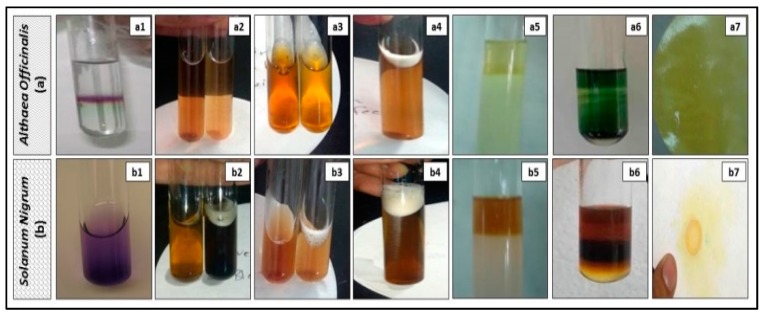
Pictures showing results of preliminary phytochemical screening of (**a**) *Althaea officinalis* flower extract in hot water, and (**b**) *Solanum nigrum* fruit extract in 99% ethanol. Where: (**a1**,**b1**): for carbohydrates, (**a2**,**b2**): for tannins, (**a3**,**b3**): for flavonoids, (**a4**,**b4**): for saponins, (**a5**,**b5**): for anthraquinone glycosides, (**a6**,**b6**): for sterols and triterpenoids, and (**a7**,**b7**): for alkaloids.

Preliminary phytochemical analysis for *Althaea officinalis* extract was positive for carbohydrates, traces of tannins, flavonoids, traces of saponins, and volatile oils. Preliminary phytochemical analysis for *Solanum nigrum* extract was positive for carbohydrates, tannins, flavonoids, saponins, sterols and triterpenoids and alkaloids (Figure 1 and Table 1).

### 3.2. Evaluation of the In Vitro Anti-Oxidant Activity (DPPH Assay)

**Figure 2:**
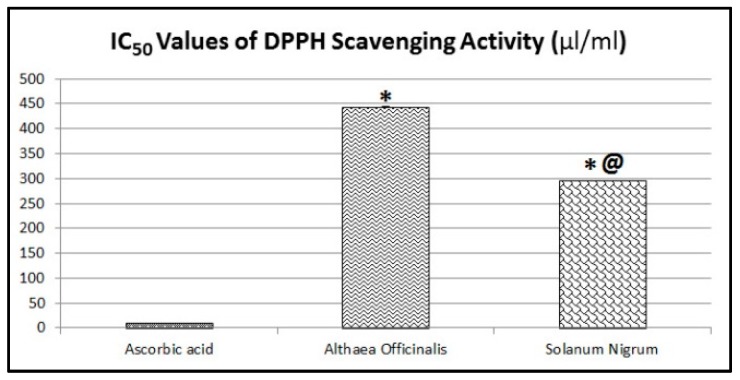
In vitro anti-oxidant activity of aqueous extracts of *Althaea officinalis* flower and ethanolic extracts of *Solanum nigrum* fruit. Each value signifies the mean ± standard error of the mean. Statistical analysis was performed via one-way ANOVA test, and after that the Tukey–Kramer multiple comparisons test was done. Where: * indicates a significant difference from ascorbic acid (*p* < 0.05), and @ indicates a significant difference from *Althaea officinalis* (*p* < 0.05).

Aqueous extracts of *Althaea officinalis* and ethanolic extracts of *Solanum nigrum* showed free radical scavenging activities against DPPH. The ethanolic extract of *Solanum nigrum* was the more active as it shows the smallest IC_50_ values. The aqueous extract of *Althaea officinalis* was the least active as it shows the highest IC_50_ values. The ethanolic extract of *Solanum nigrum* (IC_50_ = 295.84 ± 0.11 µg/mL) showed a significant difference when compared to the standard ascorbic acid (IC_50_ = 10 ± 0.03 µg/mL). The aqueous extract of *Althaea officinalis* (IC_50_ = 443.60 ± 0.39 µg/mL) showed a significant difference when compared to the standard ascorbic acid and ethanolic extract of *Solanum nigrum* (Figure 2).

### 3.3. Macroscopic Examination (Assessment of Gross Mucosal Damage) (Ulcer Number, Ulcer Index, and Preventive Index)

**Figure 3:**
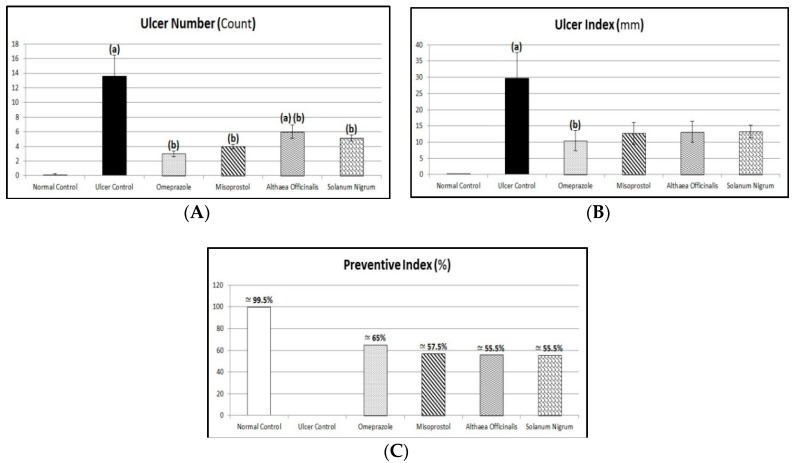
Protective effects of *Althaea officinalis* and *Solanum nigrum* compared to the standards misoprostol and omeprazole on ulcer number (**A**), ulcer index (**B**), and preventive index (**C**) against pyloric-ligation/indomethacin-induced gastric-ulceration experimentally in male rats. Each value signifies the mean of 6–8 animals ± standard error of the mean. Statistical analysis was performed via a one-way ANOVA test, and after that a Tukey–Kramer multiple comparisons test was done. Where: ^(a)^ indicates a significant difference from normal control group (*p* < 0.05), and ^(b)^ indicates a significant difference from gastric ulcer control group (*p* < 0.05).

Exposing rats to pyloric-ligation/indomethacin administration (gastric ulcer control group) resulted in significant ulceration in the glandular part of their stomachs as it expressed elevations in ulcer number and ulcer index of 13.57 ± 2.89 and 29.71 ± 7.85, respectively, in comparison to the normal control group. Omeprazole pretreatment expressed a significant reduction in ulcer number and ulcer index, to 22.11% and 35.09%, respectively, in comparison to the gastric ulcer control group, providing 64.90% protection against gastric mucosal injury. Similarly, misoprostol pretreatment expressed a significant reduction in ulcer number and ulcer index, to 29.48% and 42.79%, respectively, in comparison to the gastric ulcer control group, providing 57.21% protection. In addition, *Althaea officinalis* pretreatment expressed a significant reduction in ulcer number and ulcer index, to 44.21% and 44.23%, respectively, in comparison to the gastric ulcer control group, providing 55.77% protection. *Solanum nigrum* pretreatment expressed a significant reduction in ulcer number and ulcer index, to about 37.89% and 44.71%, respectively, in comparison to the gastric ulcer control group, providing 55.29% protection (Figure 3).

### 3.4. Microscopic Examination (Routine H and E Staining and Alcian Blue Staining)

#### 3.4.1. Routine H and E Staining (Histopathological Examination of Stomach)

**Figure 4:**
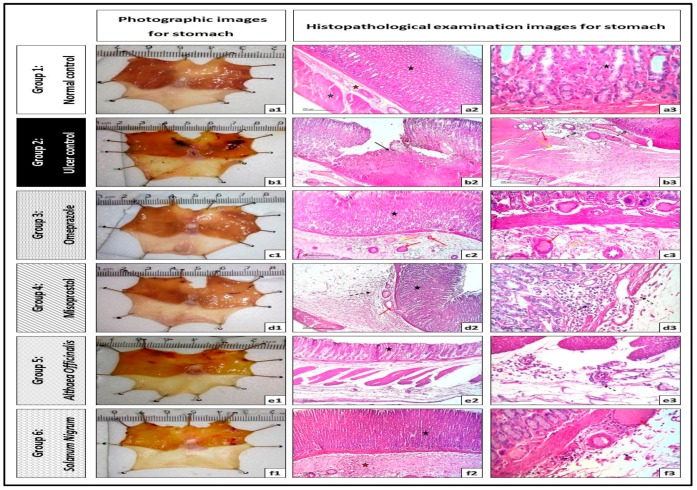
Photographic image from stomachs and histopathological examination image (50 µm–200 µm magnification) of each group. Where: (**a1**–**a3**): normal control group, (**b1**–**b3**): gastric ulcer control group (pyloric-ligation/indomethacin-induced gastric-ulceration), (**c1**–**c3**): omeprazole (20 mg/kg, p.o.) group, (**d1**–**d3**): misoprostol (300 µg/kg, p.o.) group, (**e1**–**e3**): *Althaea officinalis* (100 mg/kg, p.o.) group, (**f1**–**f3**): *Solanum nigrum* (200 mg/kg, p.o.) group. Where: (black star) is gastric mucosa including glandular tissue, (red star) is the submucosal layer, (green star) is the muscular coat, (black arrow) is destructed cellular elements, (yellow arrow) is edema, (red arrow) is congested or dilated blood vessels, and (dashed arrow) is inflammatory cell infiltrations.

As shown in Figure 4 and Table 2, normal rats’ stomachs showed normal histological structures of gastric mucosa including glandular tissue, submucosa, muscular coat, and covering serosa without abnormal alterations (Figure 4a1,a2,a3). Exposing rats to pyloric-ligation/indomethacin administration (gastric ulcer control rats) demonstrated multiple ulcerations and necrotic foci in gastric mucosa with destructed cellular elements and extensive submucosal edema accompanied with congested and dilated blood vessels with many inflammatory cells infiltrations (Figure 4b1,b2,b3). Pretreatment with omeprazole showed apparent intact glandular mucosa. However, extensive submucosal edema is evident with congested blood vessels. Minimal inflammatory cells infiltrations were recorded (Figure 4c1,c2,c3). Similarly, misoprostol pretreatment is almost like the omeprazole (1st standard pretreatment) group records. However, scattered focal aggregations of inflammatory cells were recorded in deep mucosal and submucosal layers (Figure 4d1,d2,d3). In addition, *Althaea officinalis* pretreatment showed apparent intact glandular mucosa structures. Mild submucosal edemas with focal perivascular aggregation of inflammatory cells were observed in the subserosal layer (Figure 4e1,e2,e3). *Solanum nigrum* pretreatment demonstrated apparent intact mucosa and glandular structures, extensive submucosal edema, congestions of blood vessels, and focal aggregation of mononuclear inflammatory cells (Figure 4f1,f2,f3).

#### 3.4.2. Alcian Blue Staining for Determination of Gastric Wall Mucus (Mucin Content)

**Figure 5:**
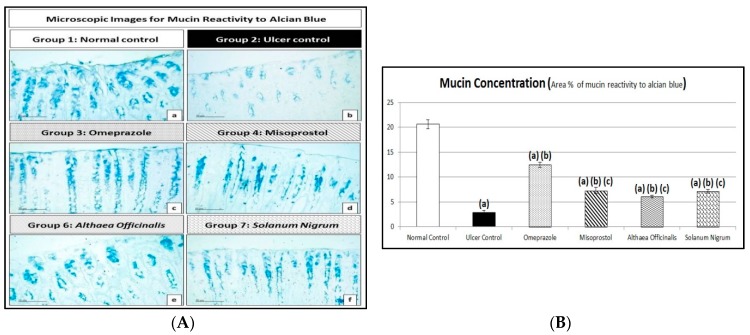
(**A**) Photomicrographs (50 µm magnification) of each group of gastric mucosa sections stained with alcian blue. Where: (**a**): normal control group, (**b**): gastric ulcer control group (pyloric-ligation/indomethacin-induced gastric-ulceration), (**c**): omeprazole (20 mg/kg, p.o.) group, (**d**): misoprostol (300 µg/kg, p.o.) group, (**e**): *Althaea officinalis* (100 mg/kg, p.o.) group, (**f**): *Solanum nigrum* (200 mg/kg, p.o.) group. All data and micrographs were obtained using a full HD microscopic camera operated by the Leica application module for tissue sections analysis (Leica Biosystems, Germany). (**B**) Protective effects of *Althaea officinalis* and *Solanum nigrum* with standards misoprostol and omeprazole on mucin content against pyloric-ligation/indomethacin-induced gastric-ulceration experimentally in male rats. Each value signifies the mean of 6–8 animals ± standard error of the mean. Statistical analysis was performed via one-way ANOVA test, and after that the Tukey–Kramer multiple comparisons test was done. Where: ^(a)^ indicates a significant difference from the normal control group (*p* < 0.05), ^(b)^ indicates a significant difference from the gastric ulcer control group (*p* < 0.05), and ^(c)^ indicates a significant difference from the omeprazole (1st standard pretreatment) group (*p* < 0.05).

Exposing rats to pyloric-ligation/indomethacin administration (gastric ulcer control group) expressed a significant reduction in mucin content of 2.9 ± 0.37% of alcian blue +ve mucin, in comparison to the normal control group. Omeprazole pretreatment significantly increased mucin content to 431.03% in comparison to the gastric ulcer control group. Misoprostol pretreatment significantly increased mucin content to 250.59%, in comparison to the gastric ulcer control group. It significantly decreased mucin content to 58.14%, in comparison to the omeprazole (1st standard pretreatment) group. In addition, *Althaea officinalis* pretreatment significantly increased mucin content to 211.21%, in comparison to the gastric ulcer control group. It significantly decreased mucin content to 49%, in comparison to the omeprazole (1st standard pretreatment) group. *Solanum nigrum* pretreatment significantly increased mucin content to 245.52%, in comparison to the gastric ulcer control group. It significantly decreased mucin content to 56.96%, in comparison to the omeprazole (1st standard pretreatment) group (Figure 5).

### 3.5. Acid Productivity Parameters (Gastric Volume, Titratable Acidity, and Acid Output Determination)

Exposing rats to pyloric-ligation/indomethacin administration (gastric ulcer control group) resulted in a significant increase in gastric volume and acid output of 4.29 ± 0.36 mL/4 h and 112.33 ± 11.73 µEq/h, respectively, in comparison to 0.18 ± 0.04 mL/4 h and 10.5 ± 0 µEq/h, respectively, for the normal control group. Alternatively, induction caused a reduction in titratable acidity, reaching a value of 105 ± 5.40 mEq/L in comparison to 140 ± 0 mEq/L for the normal control group.

The standard and test pretreatment groups showed significantly increased gastric volume and acid output in comparison to the normal control group, but they had a reduction in titratable acidity in comparison to the normal control group.

**Figure 6:**
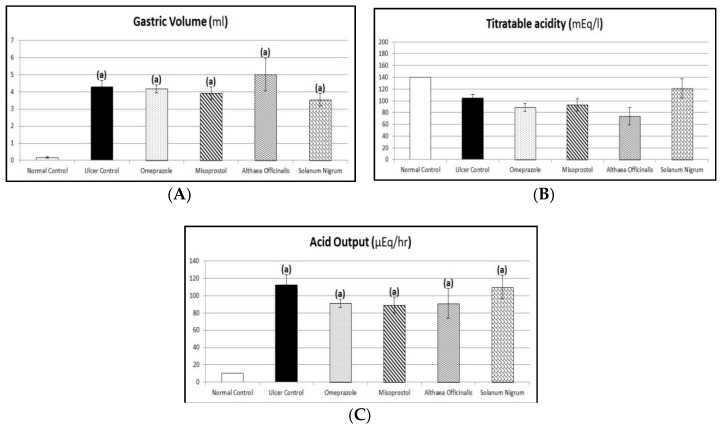
Protective effects of *Althaea officinalis* and *Solanum nigrum* with standards misoprostol and omeprazole on gastric volume (**A**), titratable acidity (**B**), and acid output (**C**) against pyloric-ligation/indomethacin-induced gastric-ulceration experimentally in male rats. Each value signifies the mean of 6–8 animals ± standard error of the mean. Statistical analysis was performed via one-way ANOVA test, and after that the Tukey–Kramer multiple comparisons test was done. Where: ^(a)^ indicates a significant difference from the normal control group (*p* < 0.05).

Omeprazole pretreatment caused change (but not significantly) in gastric volume, titratable acidity, or acid output to 4.17 ± 0.24 mL/4 h, 88.75 ± 6.45 mEq/L, and 90.98 ± 4.50 µEq/h, respectively, in comparison to the gastric ulcer control group. Similarly, misoprostol pretreatment caused change (but not significantly) in gastric volume, titratable acidity, or acid output to 3.91 ± 0.37 mL/4 h, 93.57 ± 10.55 mEq/L, and 88.74 ± 9.05 µEq/h, respectively, in comparison to the gastric ulcer control group. In addition, *Althaea officinalis* pretreatment caused change (but not significantly) in gastric volume, titratable acidity, or acid output to 5 ± 0.96 mL/4 h, 74.17 ± 14.63 mEq/L, and 90.76 ± 17.19 µEq/h, respectively, in comparison to the gastric ulcer control group. *Solanum nigrum* pretreatment caused change (but not significantly) in gastric volume, titratable acidity, or acid output to 3.53 ± 0.36 mL/4 h, 121 ± 16.21 mEq/L, and 109.69 ± 13.50 µEq/h, respectively, in comparison to the gastric ulcer control group. Also, the test pretreatment groups did not significantly change gastric volume, titratable acidity, or acid output in comparison to the omeprazole (1st standard pretreatment) group or the misoprostol (2nd standard pretreatment) group (Figure 6).

### 3.6. Assessment of Aggressive Factors (Peptic Activity in Gastric Secretions, Gastric Mucosal Histamine, and Plasma Gastrin Contents)

**Figure 7:**
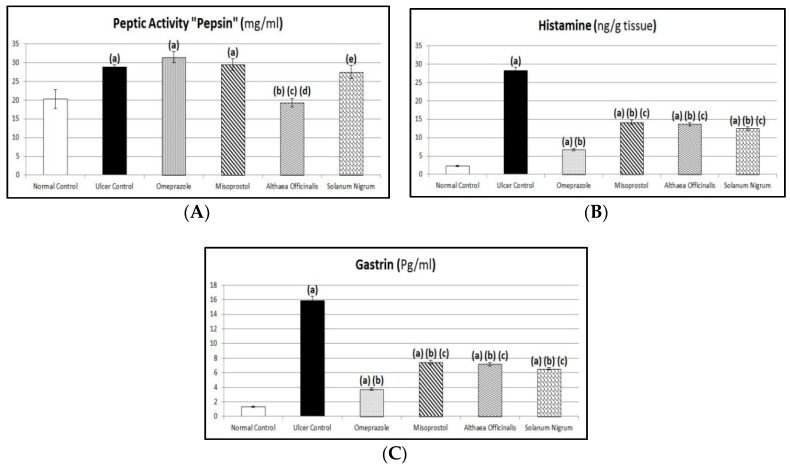
Protective effects of *Althaea officinalis* and *Solanum nigrum* with standards misoprostol and omeprazole on peptic activity (**A**), histamine (**B**), and gastrin (**C**) against pyloric-ligation/indomethacin-induced gastric-ulceration experimentally in male rats. Each value signifies the mean of 6–8 animals ± standard error of the mean. Statistical analysis was performed via one-way ANOVA test, and after that the Tukey–Kramer multiple comparisons test was done. Where: ^(a)^ indicates a significant difference from the normal control group (*p* < 0.05), ^(b)^ indicates a significant difference from the gastric ulcer control group (*p* < 0.05), ^(c)^ indicates a significant difference from the omeprazole (1st standard pretreatment) group (*p* < 0.05), and ^(d)^ indicates a significant difference from the misoprostol (2nd standard pretreatment) group (*p* < 0.05).

Exposing rats to pyloric-ligation/indomethacin administration (gastric ulcer control group) expressed a significant elevation in peptic activity, histamine, and gastrin contents as they were 28.77 ± 0.62 mg/mL, 28.13 ± 0.93 ng/g, and 15.85 ± 0.64 Pg/mL, respectively, in comparison to 20.27 ± 2.49 mg/mL, 2.23 ± 0.14 ng/g, and 1.33 ± 0.09 Pg/mL, respectively, for the normal control group. Omeprazole pretreatment did not significantly affect peptic activity as it was 31.39 ± 1.47 mg/mL in comparison to the gastric ulcer control group. But, it expressed a significant reduction in histamine and gastrin contents to 24% and 23.5%, respectively, in comparison to the gastric ulcer control group. Similarly, misoprostol pretreatment did not significantly affect peptic activity as it was 29.50 ± 1.55 mg/mL in comparison to the the gastric ulcer control group. But, it expressed a significant reduction in histamine and gastrin contents to 50.49% and 46.85%, respectively, in comparison to the gastric ulcer control group. Also, it expressed a significant elevation in histamine and gastrin contents to 210.37% and 199.33%, respectively, in comparison to the omeprazole (1st standard pretreatment) group. In addition, *Althaea officinalis* pretreatment expressed a significant reduction in peptic activity, histamine, and gastrin contents to 67.08%, 48.53%, and 45.27%, respectively, in comparison to the gastric ulcer control group and it expressed a significant reduction in peptic activity to 61.48%, in comparison to the omeprazole (1st standard pretreatment) group. Also, it expressed a significant reduction in peptic activity to 65.42% in comparison to the misoprostol (2nd standard treatment) group. It expressed a significant elevation in histamine and gastrin contents to 202.22% and 192.62%, respectively, in comparison to the omeprazole (1st standard pretreatment) group. *Solanum nigrum* pretreatment did not significantly affect peptic activity as it was 27.45 ± 1.82 mg/mL in comparison to the gastric ulcer control group. But, it expressed a significant reduction in histamine and gastrin contents to 44.27% and 41.17%, respectively, in comparison to the gastric ulcer control group. Also, it expressed a significant elevation in histamine and gastrin contents to 184.44% and 175.17%, respectively, in comparison to the omeprazole (1st standard pretreatment) group. Also, it expressed a significant elevation in peptic activity to 142.23% in comparison to the *Althaea officinalis* (1st test pretreatment) treated group (Figure 7).

### 3.7. Oxidative Stress Biomarkers (Gastric Mucosal NO, GSH, and MDA Contents and SOD Activity)

**Figure 8:**
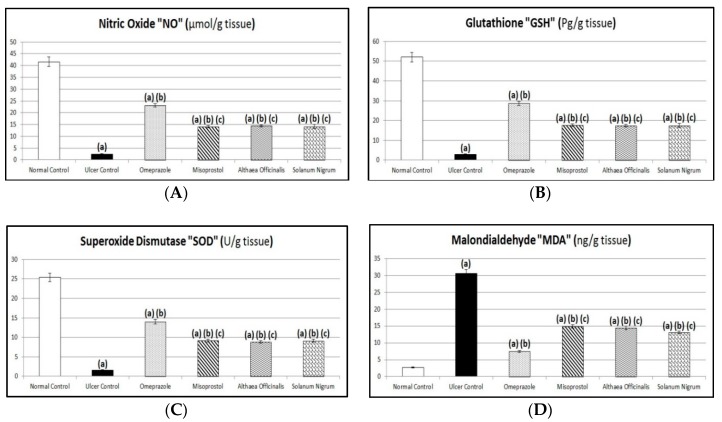
Protective effects of *Althaea officinalis* and *Solanum nigrum* with standards misoprostol and omeprazole on nitric oxide “NO” (**A**), glutathione “GSH” (**B**), superoxide-dismutase “SOD” (**C**), and malondialdehyde “MDA” (**D**) against pyloric-ligation/indomethacin-induced gastric-ulceration experimentally in male rats. Each value signifies the mean of 6–8 animals ± standard error of the mean. Statistical analysis was performed via one-way ANOVA test, and after that the Tukey–Kramer multiple comparisons test was done. Where: ^(a)^ indicates a significant difference from the normal control group (*p* < 0.05), ^(b)^ indicates a significant difference from the gastric ulcer control group (*p* < 0.05), and ^(c)^ indicates a significant difference from the omeprazole (1st standard pretreatment) group (*p* < 0.05).

Exposing rats to pyloric-ligation/indomethacin administration (gastric ulcer control group) expressed a significant reduction in GSH and NO contents and SOD activity as it was 2.83 ± 0.34 Pg/g, 2.40 ± 0.26 µmol/g and 1.58 ± 0.17 U/g, respectively, in comparison to 51.98 ± 2.45 Pg/g, 41.63 ± 1.95 µmol/g, and 25.38 ± 1.04 U/g, respectively, for the normal control group and it expressed a significant elevation in MDA content to 30.50 ± 1.22 ng/g, in comparison to 2.75 ± 0.17 ng/g for the normal control group. Omeprazole pretreatment expressed a significant elevation in GSH and NO contents and SOD activity to 1014.16%, 962.50%, and 892.06%, respectively, in comparison to the gastric ulcer control group. It expressed a significant reduction in MDA content to 24.51% in comparison to the gastric ulcer control group. Misoprostol pretreatment expressed a significant elevation in GSH and NO contents and SOD activity to 624.78%, 589.58%, and 580.95%, respectively, in comparison to the gastric ulcer control group. It expressed a significant reduction in GSH and NO contents and SOD activity to 61.61%, 61.26%, and 65.13%, respectively, in comparison to the omeprazole (1st standard pretreatment) group. It expressed a significant reduction in MDA content to 48.85% in comparison to the gastric ulcer control group. It expressed a significant elevation in MDA content to 199.33% in comparison to the omeprazole (1st standard pretreatment) group. In addition, *Althaea officinalis* pretreatment expressed a significant elevation in GSH and NO contents and SOD activity to 617.70%, 602.08%, and 561.91%, respectively, in comparison to the gastric ulcer control group. It expressed a significant reduction in GSH and NO contents and SOD activity to 60.91%, 62.55%, and 62.99%, respectively, in comparison to the omeprazole (1st standard pretreatment) group. It expressed a significant reduction in MDA content to 47.29% in comparison to the gastric ulcer control group. It expressed a significant elevation in MDA content to 192.98% in comparison to the omeprazole (1st standard pretreatment) group. *Solanum nigrum* pretreatment expressed a significant elevation in GSH and NO contents and SOD activity to 617.70%, 589.58%, and 579.37%, respectively, in comparison to the gastric ulcer control group. It expressed a significant reduction in GSH and NO contents and SOD activity to 60.91%, 61.26%, and 64.95%, respectively, in comparison to the omeprazole (1st standard pretreatment) group. It expressed a significant reduction in MDA content to 42.79% in comparison to the gastric ulcer control group. It expressed a significant elevation in MDA content to 174.58% in comparison to the omeprazole (1st standard pretreatment) group (Figure 8).

### 3.8. Protective Factors (Gastric Mucosal PG-E_2_ and PG-I_2_ Contents)

Exposing rats to pyloric-ligation/indomethacin administration (gastric ulcer control group) expressed a significant reduction in PG-E_2_ content as it was 1.98 ± 0.16 Pg/g in comparison to 38.65 ± 1.62 Pg/g for the normal control group. Omeprazole pretreatment expressed a significant elevation in PG-E_2_ content to 1022.78% in comparison to the gastric ulcer control group. Misoprostol pretreatment expressed a significant elevation in PG-E_2_ content to 564.56% in comparison to the gastric ulcer control group. It expressed a significant reduction in PG-E_2_ content to 55.2% in comparison to the omeprazole (1st standard pretreatment) group. In addition, *Althaea officinalis* pretreatment expressed a significant elevation in PG-E_2_ content to 578.48% in comparison to the gastric ulcer control group. It expressed a significant reduction in PG-E_2_ content to 56.56% in comparison to the omeprazole (1st standard pretreatment) group. *Solanum nigrum* pretreatment expressed a significant elevation in PG-E_2_ content to 573.42% in comparison to the gastric ulcer control group. It expressed a significant reduction in PG-E_2_ content to 56.06% in comparison to the omeprazole (1st standard pretreatment) group.

The standard and test pretreatment groups increased PG-I_2_ content to different values in comparison to the gastric ulcer control group

**Figure 9:**
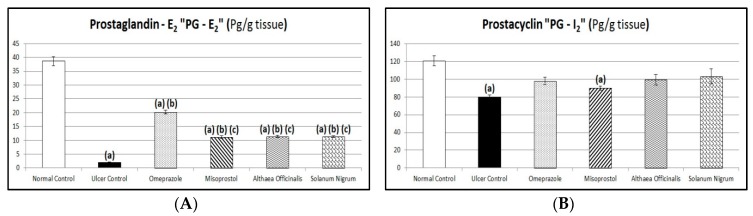
Protective effects of *Althaea officinalis* and *Solanum nigrum* with standards misoprostol and omeprazole on prostaglandin-E_2_ “PG-E_2_” (**A**) and prostacyclin “PG-I_2_” (**B**) against pyloric-ligation/indomethacin-induced gastric-ulceration experimentally in male rats. Each value signifies the mean of 6–8 animals ± standard error of the mean. Statistical analysis was performed via one-way ANOVA test, and after that the Tukey–Kramer multiple comparisons test was done. Where: ^(a)^ indicates a significant difference from the normal control group (*p* < 0.05), ^(b)^ indicates a significant difference from the gastric ulcer control group (*p* < 0.05), and ^(c)^ indicates a significant difference from the omeprazole (1st standard pretreatment) group (*p* < 0.05).

Exposing rats to pyloric-ligation/indomethacin administration (gastric ulcer control group) showed a significant decrease in PG-I_2_ content as it was 79.68 ± 2.59 Pg/g in comparison to 120.53 ± 5.48 Pg/g for the normal control group. Pretreatment with omeprazole, misoprostol, *Althaea officinalis,* and *Solanum nigrum* increased (but not significantly) PG-I_2_ content as it was 97.93 ± 4.28 Pg/g, 89.85 ± 2.32 Pg/g, 99.23 ± 5.66 Pg/g, and 102.98 ± 8.31 Pg/g, respectively, in comparison to the gastric ulcer control group (Figure 9).

### 3.9. Immunological Markers (Gastric Mucosal TNF-α and IL-1β Contents and CBS and HO-1 Activities) Estimated by Western Blot Analysis

**Figure 10:**
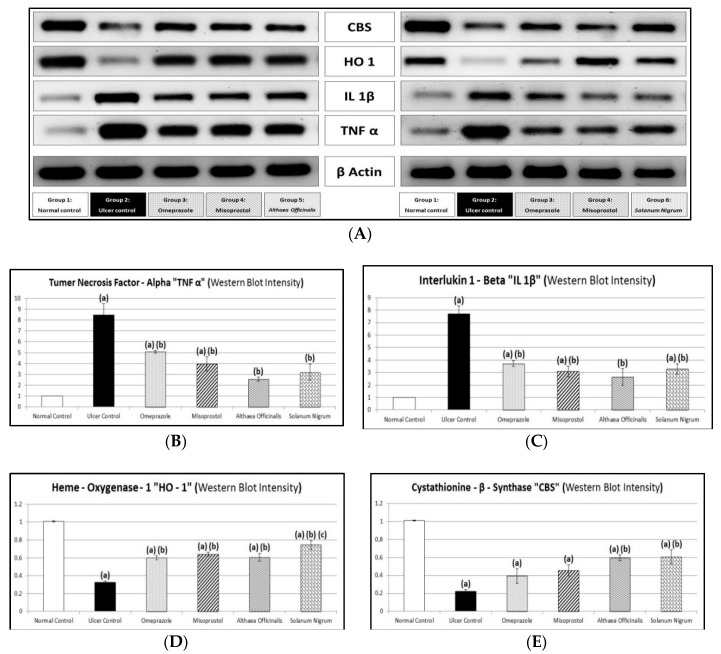
Western blot analysis (**A**) and figure showing protective effects of *Althaea officinalis* and *Solanum nigrum* extracts compared to standards misoprostol and omeprazole on tumor-necrosis-factor-alpha “TNF-α” (**B**), interleukin-1-beta “IL-1β” (**C**), heme-oxygenase-1 “HO-1” (**D**), and cystathionine-β-synthase “CBS” (**E**) against pyloric-ligation/indomethacin-induced gastric-ulceration experimentally in male rats. Each value signifies the mean of 6–8 animals ± standard error of the mean. Statistical analysis was performed via one-way ANOVA test, and after that the Tukey–Kramer multiple comparisons test was done. Where: ^(a)^ indicates a significant difference from the normal control group (*p* < 0.05), ^(b)^ indicates a significant difference from the gastric ulcer control group (*p* < 0.05), and ^(c)^ indicates a significant difference from the omeprazole (1st standard pretreatment) group (*p* < 0.05).

Gastric mucosal TNF-α and IL-1β contents in the normal control group was 1 ± 0 and 1.01 ± 0.01, respectively, and gastric mucosal CBS and HO-1 activities in the normal control group was 1.01 ± 0.01 and 1.01 ± 0.01, respectively. Exposing rats to pyloric-ligation/indomethacin administration showed a significant increase in TNF-α and IL-1β contents to 847.5% and 763.89%, respectively, in comparison to normal control rats. On other hand, it showed a significant decrease in CBS and HO-1 activities to 22.12% and 32.14%, respectively, in comparison to the normal control group. Omeprazole pretreatment significantly decreased TNF-α and IL-1β contents to 59.88% and 48.38%, respectively, in comparison to the gastric ulcer control. It did not significantly affect CBS activity as it was 0.39 ± 0.08. But it significantly increased HO-1 activity to 185.76% in comparison to the gastric ulcer control group. Misoprostol pretreatment significantly decreased TNF-α and IL-1β contents to 46.90% and 40.26%, respectively, in comparison to the gastric ulcer control group. On other hand, it did not significantly affect CBS activity as it was 0.46 ± 0.06. But it significantly increased HO-1 activity to 198.142% in comparison to the gastric ulcer control group. Additionally, *Althaea officinalis* pretreatment significantly decreased TNF-α and IL-1β contents to 30.38% and 34.42%, respectively, in comparison to the gastric ulcer control group. But, it significantly increased CBS and HO-1 activities to 266.82% and 187.31%, respectively, in comparison to the gastric ulcer control group. *Solanum nigrum* pretreatment significantly decreased TNF-α and IL-1β contents to 37.76% and 42.53%, respectively, in comparison to the gastric ulcer control group. It significantly increased CBS and HO-1 activities to 271.3% and 230.65%, respectively, in comparison to the gastric ulcer control group. It significantly increased HO-1 activity to 124.17%, in comparison to the omeprazole (1st standard pretreatment) group (Figure 10).

## 4. Discussion

In the present study, the gastro-protective activity and the anti-oxidant effect of *Althaea officinalis* and *Solanum nigrum* extracts was investigated in pyloric-ligation/indomethacin-induced gastric-ulceration in male rats and their possible underlying mechanisms included in their actions were investigated.

Preliminary phytochemical analysis of test extracts (aqueous extracts of *Althaea officinalis* and ethanolic extracts of *Solanum nigrum*) was accomplished prior to the pharmacological study. The *Althaea officinalis* flower extract was positive for carbohydrates, traces of tannins, flavonoids, traces of saponins, and volatile oils. In agreement, Al-Snafi [60] has documented similar results. *Solanum nigrum* fruit extract was positive for carbohydrates, tannins, flavonoids, saponins, sterols and triterpenoids and alkaloids, in agreement with the results obtained by El-Hawary et al. and Rani et al. [37,61].

The inhibition of gastric ulcers induced by indomethacin is believed to be mediated by anti-oxidant effects [8,62]. Free radical scavenging drugs, either from synthetic or herbal sources, have an important role in decreasing, de-activating, and eliminating reactive oxygen species (ROS). Accordingly, these drugs exert a significant protection for tissues from oxidative damage as well as enhancement of wound healing [63]. Many previous investigations documented the major role of oxidative stress in the pathogenesis of many diseases, including gastric ulcers [64]. Oxidative stress is considered one of the major causative factors of gastric ulcers. In the normal state, reactive oxygen species (ROS) are always produced, but eliminated by anti-oxidant defense mechanisms of the body. Imbalance in the anti-oxidant defense system resulted in the generation of gastric damage [65]. Oxidative damage, cell death, and epithelial damage resulted from increased ROS. Additionally, it was documented that ROS expressed an elevation in gastric acid secretion by releasing histamine and reducing mucus content by diminishing prostaglandin synthesis [33].

Aqueous extracts of *Althaea officinalis* and ethanolic extracts of *Solanum nigrum* showed free radical scavenging activities against DPPH. The results of the in vitro anti-oxidant activity of test extracts were in agreement with previous studies [32,60,66,67,68]. Ethanolic extracts of *Solanum nigrum* showed the highest anti-oxidant effect, exhibiting the smallest IC_50_ values. Alternatively, the aqueous extract of *Althaea officinalis* was the least active by showing the highest IC_50_ values.

Non-steroidal anti-inflammatory drugs (NSAIDs) are considered one of the most frequently used medication classes worldwide. NSAIDs administration represents the main reason for gastric-ulceration [18]. Indomethacin, in particular, is commonly used and considered a clinically appropriate experimental model for gastric ulcer induction. Mechanistically, indomethacin generates gastric ulcers by inhibiting prostaglandin synthesis, generating reactive oxygen species (ROS) production, initiating lipid peroxidation formation [8,69], prompting apoptosis and necrosis of stomach cells [70,71], reducing bicarbonate and mucus secretion, increasing gastric motility [72], elevating pro-inflammatory cytokines production [73], and finally, disrupting nitric oxide production in gastric tissues [74,75]. The ulcerogenic mechanism of indomethacin is complex and remains unclear [76]. Briefly, indomethacin-induced gastric-ulceration was suggested to be caused by inhibiting the release of protective factors, accumulating aggressive factors, and aggravating oxidant parameters while diminishing anti-oxidant parameters [8,77]. Accordingly, the use of drugs with anti-oxidant activity, and the capability to modify nitric oxide content, can protect against indomethacin-induced gastric-ulceration [75].

Pyloric-ligation induced gastric-ulceration resulted from auto-digestion and break down of the stomach wall [78] by the digestive effect of accumulated gastric juice (gastric acid and pepsin), interference of gastric blood circulation [33,79], and increased production of free radicals [12]. Drugs which express a reduction in gastric juice secretion and elevation in mucus production can prevent the pyloric-ligation induced gastric-ulcerations [80].

Proton-pump inhibitors (PPIs) are often co-administered with NSAIDs to reduce NSAID-induced gastrointestinal (GI) adverse events. This co-administration is generally regarded as safe, and is included in many of the guidelines on NSAID prescription [81].

Misoprostol had been used in many investigations as a positive control drug [82]. Previously, Adinortey et al. reported that it is advisable to compare the potential drug or test material with cytoprotectant reference drugs such as misoprostol that are known to prevent peptic ulcers [22]. Misoprostol is effective but its side effects limit its use [21]. Misoprostol protects the GI mucosa by stimulating mucus/bicarbonate secretion [81].

Some herbs have shown inhibition of gastric mucosal damage induced experimentally by necrotizing agents through their anti-secretory and anti-oxidant properties [21].

*Althaea officinalis* is traditionally used as a treatment for the irritation of mucous membranes, including use as a gargle for mouth and throat ulcers and gastric ulcers [60,83]. *Althaea officinalis* flower is commonly used in folk medicine in Middle East countries. This flower contains a variety of bioflavonoids, vitamins, and anti-oxidant compounds [35]. The extract of *Althaea officinalis* exhibited strong anti-oxidant activity in different anti-oxidant tests [32]. The aqueous extract of *Althaea officinalis* was demonstrated to be potentially helpful in treating gastric ulcers with no visible adverse effects [25,34,35,84]. The observed gastro-protective effect of *Althaea officinalis* could be attributed to active phytoconstituents found in the extract such as flavonoids and mucilage polysaccharides [25]. *Althaea officinalis* has a mucus protection (cytoprotection effect) and an anti-oxidant effect [85]. These results are also supported by and continuous to our previous research on aqueous extracts of *Althaea officinalis* flowers [86].

*Solanum nigrum* is a herbal plant that has been traditionally used in oriental medicines and is believed to have various biological activities [5,66,67,87]. *Solanum nigrum* is currently used in Egyptian folk medicine for some of gastro-intestinal tract (GIT) disorders [17]. Although *Solanum nigrum* is a rich source of one of the plants’ most dreaded toxins [88], the fruit of *Solanum nigrum* is reported to have anti-oxidant, cytoprotective, anti-ulcerogenic, ulcer healing, anti-inflammatory, and anti-tumor effects in rats [1,67,87]. Additionally, it is a potential herbal alternative that acts as an anti-cancer agent [61]. Many researchers have proven that anti-oxidants may play an important role not only by protecting against gastric mucosal injury, but also by inhibiting progression of gastric ulcers. The observed cytoprotective and anti-oxidative activity of *Solanum nigrum* extract is attributed to the presence of biologically active phytoconstituents having anti-oxidative nature [5].

Results of the current investigation revealed that pyloric ligation of rat stomachs followed by administration of indomethacin (25 mg/kg, i.p.) caused significant ulceration in the glandular area and degenerative changes in gastric tissues of the rat stomach as seen in histopathological examination as it showed epithelial loss, congested blood vessels with inflammatory cell infiltration, associated with considerable elevation in ulcer number and ulcer index, which was in agreement with previous studies that indicated that NSAIDs like indomethacin can generate observable gastric ulcers in experimental animals [1,6,18,39,75,89,90]. Pyloric-ligation/indomethacin-induced mucosal injury is attributed to various processes, including infiltration of leukocytes, inhibition of PG-E_2_, initiation of lipid peroxidation, decreasing levels of anti-oxidants, and induction of apoptosis [91,92].

Pretreatment with omeprazole protected animals from pyloric-ligation/indomethacin-induced gastric-ulceration as manifested by significantly reduced ulcer number and ulcer index. Similar results were obtained by Bhalke et al., who demonstrated that omeprazole in a dose of 20 mg/kg produced a significant reduction in the ulcer index and has high protection against aspirin-induced gastric ulcers and produced protection against the pylorus-ligation induced gastric ulcers model [7]. In another study, omeprazole for three days reduced the number of inflammatory cells and mucosal congestion, and increased the number of healthy normal cells in the gastric mucosa, submucosa, serosa, and muscle layers [93]. The reference drug omeprazole showed protection against aspirin and pyloric-ligation induced ulcer models [94].

Additionally, misoprostol protected animals from pyloric-ligation/indomethacin-induced gastric-ulceration as manifested by significantly reduced ulcer number and ulcer index. Similar results were obtained by Bhalke et al., who demonstrated that misoprostol has shown a significant effect on the indomethacin-induced ulcer model with a high protection index [7]. Moreover, misoprostol administered before indomethacin significantly reduced the mean ulcer index in comparison to the mean ulcer index value of indomethacin intoxicated rats [95]. In agreement with our results, the histopathological examination of the stomach in the rats treated by misoprostol before indomethacin showed normal mucosal membrane adjacent to areas of gastritis [95].

Additionally, *Althaea officinalis* extract pretreatment showed anti-ulcer activity as observed by a significant reduction of the ulcer number and ulcer index after pyloric-ligation/indomethacin administration. In a previous study, it was shown that mucilage and flavonoids have the property of covering and protecting the gastric mucosa, thereby reducing the incidence of gastric ulcers [25]. Animals receiving *Althaea officinalis* extract showed a dose-dependent protection against ethanol-induced ulcers [96].

According to our study, *Solanum nigrum* extract significantly reduced the ulcer number and ulcer index after pyloric-ligation/indomethacin administration. Earlier, it was demonstrated that the aerial parts of *Solanum nigrum* were reported to decrease the ulcer index significantly [17]. In addition, Awaad et al. reported that oral treatment with the methanol extract of *Solanum nigrum* significantly inhibited the gastric lesions [24]. Earlier, it has been reported earlier that *Solanum nigrum* could offer an anti-ulcer action as it significantly inhibited the gastric lesions induced by cold restraint, indomethacin, pyloric-ligation, and ethanol. The reduction of ulcer size by *Solanum nigrum* was evident by histological findings [1]. Moreover, *Solanum nigrum* administered orally caused a dose-dependent decrease in the ulcer index and could inhibit the increase in area of gastric mucosal lesions in aspirin-induced ulceration in rats [5]. The *Solanum nigrum* extract decreased the mean ulcer index and proved its protective effect against ulcer formation due to pylorus-ligation [97].

Gastric mucus is the first guard against gastric acid and other ulcerogens. Mucus and bicarbonate together adhere to the epithelium working as a barrier against auto-digestion. The gastric mucus barrier has a crucial role in gastric ulcer protection. The ulcerogenic substances induce dissipation of the mucus gel layer and thus cause ulceration [26]. Previously, it was documented that gastric mucus has anti-oxidant activity, thereby reducing gastric-ulceration mediated by oxygen free radicals [19].

Our results showed that the induction of gastric ulceration by pyloric-ligation/indomethacin administration resulted in a significant decrease in mucin content in comparison to normal control rats. This result is in accordance with that obtained by previous researches [18,98].

Pretreatment with omeprazole significantly increased mucin content in comparison to the gastric ulcer control group. Similar results were obtained by previous studies [94,99]. Interestingly, animals pretreated with omeprazole showed preserved gastric mucus levels [100]. Omeprazole, a classic anti-ulcer drug, is known as not only a proton pump inhibitor, but also as a stimulator of gastric mucus secretion [8].

Misoprostol pretreatment significantly increased mucin content in comparison to the gastric ulcer control group, which was in harmony with investigations reporting that misoprostol administration before indomethacin-induced gastric-ulcerations produced an increase in mucin production [95].

In addition, *Althaea officinalis* pretreatment significantly increased mucin content in comparison to the gastric ulcer control group. Previously, the clinically proven effects were related to the presence of bioadhesive and mucilaginous polysaccharides, leading to the physical formation of a mucin-like substance on top of the irritated tissues [84].

Meanwhile, our results revealed that *Solanum nigrum* pretreatment significantly increased mucin content.

Acidity has a considerable role in the genesis of indomethacin-induced gastric-ulceration. Indomethacin-induced gastric-ulceration is magnified by high acid concentration in the stomach [101]. Histamine release results in the elevation of gastric juice secretion [18]. Gastrin is a hormone that can regulate gastric acid secretion, releases histamine, and regulates gastric endocrine cell proliferation. Over-secretion of gastrin hormone may induce hyper-secretion of acid by the parietal cells, causing gastric ulcers [1].

Significant increases in the gastric volume and acid output were noticed in ulcerated rats. Similar results have been reported by Inas et al. [18]. In addition, it was concluded that pyloric-ligation of rats for 4 h resulted in accumulation of gastric secretory volume [7]. A similar result was obtained by Adewoya and Salami who stated that indomethacin blocks synthesis of prostaglandins leading to an increased gastric acid secretion [99]. But, our results demonstrate that titratable acidity was not affected by gastric-ulceration induction.

Omeprazole did not significantly affect gastric volume, titratable acidity, or acid output in comparison to the gastric ulcer control group after pyloric-ligation/indomethacin-induced gastric-ulceration. In contrast, previous studies demonstrated that omeprazole showed a significant reduction in concentration and output of acid and pepsin secretion in pylorus-ligated rats [1]. Additionally, PPIs are known to suppress not only acid secretion but also the volume of gastric juice [102]. Moreover, the standard omeprazole treated group showed titratable acidity which is less than that of the negative (ulcer) control group [7].

Misoprostol pretreatment did not significantly change gastric volume, titratable acidity, or acid output in comparison to the gastric ulcer control group. These results were in contrast to the research which reported that misoprostol produced an increase in bicarbonate and decreased acid secretion [95,103].

Meanwhile, *Althaea officinalis* pretreatment did not significantly change gastric volume, titratable acidity, or acid output in comparison to the gastric ulcer control group.

Additionally, *Solanum nigrum* pretreatment did not significantly change gastric volume, titratable acidity, acid output, or peptic activity in comparison to the gastric ulcer control group. In contrast, previous investigations demonstrated that *Solanum nigrum* were reported to inhibit acid and pepsin secretions [17]. In another study, *Solanum nigrum* extract showed concomitant attenuation of gastric secretory volume, acidity, and pepsin secretion in ulcerated rats as in the 4 hrs pylorus-ligated rats, it decreased the gastric juice volume and reversed the increased output of acid and pepsin secretion [1].

In shay rats, administration of indomethacin (25 mg/kg, i.p.) showed an increase in peptic activity in agreement with results reported by Inas et al. and Malash et al. [18,98]. Our results showed that pyloric-ligation/indomethacin administration significantly increased gastric tissue histamine content. These results are in harmony with previous investigations concluding that indomethacin blocks synthesis of prostaglandins leading to an increased parietal cell number [99]. Additionally, gastric ulcer control rats showed a significant increase in gastrin content in comparison to normal control rats. This result is in accordance with that obtained by Jainu and Devi who demonstrated that ethanol treatment (ulcerated rats) induced a significant increase in the concentrations of plasma gastrin hormone [1].

Pretreatment with omeprazole did not significantly affect peptic activity in comparison to the gastric ulcer control group. In contrast, Kumar et al. and Jainu and Devi demonstrated that omeprazole caused significant reduction in concentration and output of pepsin secretion in pylorus-ligated rats [1,104]. Pretreatment with omeprazole was found to reduce pepsin concentration to near normal levels [105]. Anti-ulcer drugs, like ranitidine and omeprazole, act by an anti-secretory mechanism via inhibition of gastric secretion and pepsin activity [80]. Additionally, omeprazole significantly decreased histamine content in comparison to the gastric ulcer control group. Lansoprazole showed significant inhibition of H^+^/K^+^-ATPase activity when compared to rats that received ethanol [1]. A significant decrease in the parietal cell count of omeprazole pretreated rats was observed when compared with ulcerated untreated rats [99]. In addition, omeprazole significantly decreased gastrin content in comparison to the gastric ulcer control group. Pretreatment with lansoprazole caused a reduction in gastrin secretion in animals as compared with ethanol-ulcerated animals [1].

In addition, misoprostol pretreatment did not significantly affect peptic activity in comparison to the gastric ulcer control group. But it significantly decreased histamine and gastrin contents in comparison to the gastric ulcer control group.

Also, *Althaea officinalis* pretreatment significantly decreased peptic activity, histamine, and gastrin contents after gastric-ulceration induced by pyloric-ligation/indomethacin administration.

Data of the current work demonstrated that *Solanum nigrum* extract significantly decreased histamine content. Similar results were obtained demonstrating that the extract offers anti-ulcer activity by blocking acid secretion through inhibition of H^+^/K^+^-ATPase [1,24]. Furthermore, *Solanum nigrum* significantly decreased gastrin content. Similarly, results of previous studies have reported that the gastro-protective effect of *Solanum nigrum* caused suppression of gastrin release thereby decreasing the acidity of the gastric juice [1,24].

Although the inhibition of cyclooxygenase and deficiency of endogenous prostaglandin is accepted as a main mechanism implicated in indomethacin-induced gastric-ulceration, there is much evidence suggesting the involvement of oxidative stress in this pathology [69,106]. Indomethacin is recognized as an inducer of ROS in animal models, leading to mucosal injury [69]. The role of toxic oxygen free radicals in the genesis of indomethacin-induced gastric ulcers has been shown [106]. Organisms have enzymatic and non-enzymatic defense mechanisms against the toxicity and tissue damage of ROS [69]. Preventive anti-oxidants, such as SOD and GSH, can decrease the gastric mucosal damaging effect of NSAIDs [5,6,107,108]. Lipid peroxidation is the result of ROS interaction with the cell membrane, subsequently generating MDA to cause oxidative gastric damage [19]. Agents such as indomethacin initiate lipid peroxidation by functioning as oxidants and cause damage by producing ROS [109]. Indomethacin significantly increased the lipid peroxidation (MDA) and decreased the level of non-enzymatic (GSH) and the activity of enzymatic (SOD) anti-oxidants in the gastric mucosa of an indomethacin-administered rat [8,91].

Indomethacin was found to produce ulcers via increasing gastric acid secretion and decreasing NO synthesis [75,110]. NO is an endogenous defensive factor for gastric cells and exhibits gastro-protective properties against different types of aggressive agents [18]. NO is involved in the maintenance of mucosal integrity through the regulation of mucus and alkaline secretions, gastric motility and microcirculation [111], modulate acid levels and blood flow in gastric tissues, modulation of mast cell activity [112], prevent membrane lipid peroxidation [8], and promotion of PG-E_2_ synthesis [18,20,89]. Evidence exists that escalating the levels of both PG-E_2_ and NO boosts gastric mucus formation and secretion [98].

Previous studies demonstrated an increase in MDA with a decrease in anti-oxidants such as GSH and SOD in gastric mucosa of indomethacin-treated rats [6,18]. Consistent with these findings, this work revealed that administration of indomethacin significantly increased the lipid peroxidation (MDA) and decreased the level of non-enzymatic (GSH) and the activity of enzymatic (SOD) anti-oxidants in the gastric mucosa of an indomethacin-administered rat [91]. The decreased concentration of GSH is typically associated with increased lipid peroxidation, evident in the present study as increased MDA level. This was in agreement with a previous study [8,18] that showed that indomethacin-induced increases in mucosal MDA levels. According to our results, pyloric-ligation/indomethacin administration significantly decreased NO content. Similarly, previous studies obtained the same result [18,98].

Pretreatment with omeprazole significantly increased gastric GSH and NO contents after ulceration, in harmony with previous investigations, whereas omeprazole appears to replenish the GSH levels [100]. Meanwhile, in the present study, omeprazole significantly decreased MDA content and significantly increased gastric SOD activity in comparison to the gastric ulcer control group. This was in agreement with a previous study that showed that indomethacin-induced increases in mucosal MDA levels have been improved by classic anti-ulcer drugs like omeprazole [8]. Additionally, omeprazole significantly attenuated the increase of MDA, and protected the stomach from depletion of GSH in comparison to the ulcer control group [20]. The levels of SOD and CAT were significantly increased at the same time that levels of MDA were significantly decreased in pre-treated groups with omeprazole [113]. As a member of PPIs, lansoprazole has shown an anti-oxidative activity by preventing NSAID-induced increases of gastric mucosal concentrations of MPO (myeloperoxidase) and MDA and depletion of GSH concentration in animals [21].

In addition, misoprostol pretreatment significantly increased gastric NO content after pyloric-ligation/indomethacin administration, in harmony with previous investigations reporting that for the misoprostol-treated group after indomethacin-induced gastric-ulceration, NO synthesis was significantly increased [82]. According to our work results, misoprostol significantly increased gastric GSH content and gastric SOD activity in comparison to the gastric ulcer control group, while it significantly decreased MDA content in comparison to the gastric ulcer control group. In a previous investigation, it was reported that as the cytoprotective effect of misoprostol is well documented, it is not yet known if this effect involves anti-oxidant potential [103]. However, treatments by misoprostol before indomethacin significantly reduced blood and tissue MDA levels. Whether misoprostol has a direct anti-oxidant activity or the reduction in MDA results indirectly from minimizing the stressful conditions imposed by indomethacin, resulting from inhibition of indomethacin-induced gastric ulcers, needs to be clarified [95].

*Althaea officinalis* pretreatment significantly increased NO, similar results that revealed that flavonoids present in *Althaea officinalis* are anti-inflammatory and useful in maintaining healthy circulation and are protective against gastric ulcers [96]. Additionally, *Althaea officinalis* pretreatment significantly increased GSH content and SOD activity after pyloric-ligation/indomethacin administration. *Althaea officinalis* was reported to promote anti-oxidant activity in a neotetrazolium model of injury [60]. Furthermore, *Althaea officinalis* pretreatment significantly reduced ulcers associated with oxidative stress as manifested by a significant decrease in MDA in comparison to the gastric ulcer control group. Tannin content in *Althaea officinalis* (scopoletin) mimics hepatic lipid peroxidation and promotes anti-oxidants activity of superoxide-dismutase and catalase [68]. Also, it was demonstrated that flavonoids present in *Althaea officinalis* can scavenge free radicals and can inhibit lipid peroxidation [32].

Data of the current work also demonstrated that *Solanum nigrum* extract significantly increased gastric GSH and NO contents and SOD activity but significantly decreased MDA content after pyloric-ligation/indomethacin administration. These results are in harmony with those reporting that the decreased levels of anti-oxidant enzymes and increased mucosal injury were altered to near normal status upon pretreatment with *Solanum nigrum* when compared to the ulcer induced rats. So, *Solanum nigrum* may exert its gastro-protective (cytoprotective) effect by a free radical scavenging activity and inhibition of lipid peroxidation, thus suggesting its probable mechanism of action. Activities of GSH, lipid peroxides (LPO), and SOD were determined to show that *Solanum nigrum* pretreated rats maintained the protein level, anti-oxidant enzyme status, and LPO levels at near normalcy when compared to ulcerated animals. The extract increases the level of reduced GSH and decreases lipid peroxidation [5,61].

Prostaglandins (PG-E_2_ and PG-I_2_) are believed to expose gastro-protective effects by decreasing stomach acid secretion, stimulating the synthesis and secretion of mucus and bicarbonate, increasing the thickness of mucus layers, and improving the blood flow of mucosa [8,114] and the maintenance of cellular integrity in the gastric mucosa [115]. PG-E_2_ and PG-I_2_ are potent vasodilators and are involved in the maintenance of homeostasis and gastric ulcer healing [12]. Previous studies reported that PG-E_2_ acts as a potential inhibitor of TNF-α [116]. PG-I_2_ is known to increase the mucus production in superficial epithelial cells, which get inhibited with the use of NSAIDs like indomethacin [117].

Indomethacin administration to shay rats showed a significant decrease in PG-E_2_ content in comparison to normal control rats. Suleyman et al. and Inas et al. reported that indomethacin causes gastric damage by reducing PG-E_2_ levels in stomach tissue [8,18]. In addition, our results showed a significant decrease in PG-I_2_ content in ulcerated rats in comparison to normal control rats. This result is in harmony with the results of previous studies [12,117].

Pretreatment with omeprazole significantly increased PG-E_2_ content in comparison to the gastric ulcer control group. This result is in harmony with that obtained in previous investigations [94]. Pretreatment with omeprazole prevented its reduction in the gastric mucosa in comparison to the ulcer control group [20]. Treatment with omeprazole, orally, can upregulate and increase the mucosal PG-E_2_ level [116]. In contrast, pretreatment with omeprazole did not significantly affect PG-I_2_ in comparison to the gastric ulcer control group.

Misoprostol pretreatment significantly increased PG-E_2_ content in comparison to the gastric ulcer control group. Previous investigations demonstrated that misoprostol indicates a gastro-protective effect probably by a pathway that involves the synthesis of prostaglandins [115]. Additionally, treatment by misoprostol caused a significant reduction in indomethacin-induced gastric-ulceration; such a cytoprotective effect is expected from a prostaglandin pathway [95]. In addition, pretreatment with misoprostol did not significantly change PG-I_2_ in comparison to the gastric ulcer control group.

*Althaea officinalis* pretreatment significantly increased PG-E_2_ but did not significantly change PG-I_2_ in comparison to the gastric ulcer control group. In contrast, another study demonstrated that the flavonoids present in *Althaea officinalis* can improve blood circulation [32].

In addition, *Solanum nigrum* extract pretreatment increased PG-E_2_ significantly and PG-I_2_ (but not significantly) contents in comparison to gastric ulcer control group. Later, *Solanum nigrum* was able to produce a significant reduction of the gastric mucosal damage induced by indomethacin. So Jainu and Devi concluded the probable local increase in prostaglandin synthesis [1].

NSAIDs (like indomethacin) might induce the synthesis of pro-inflammatory cytokines (like TNF-α and leukotrienes as IL-1β) [91,107,116]. TNF-α and IL-1β are the key immunoregulatory cytokines that amplify the inflammatory response by activating a cascade of immune cells [1,26]. TNF-α overproduction increases the risk of gastric ulcer and cancer [18]. TNF-α also provokes its pro-inflammatory activity via increased IL-1β production, initiation of cytotoxic, apoptotic responses, leukocyte activation, and tissue infiltration [26,118].

According to our results, pyloric-ligation/indomethacin administration (25 mg/kg, i.p.) showed significantly elevated gastric TNF-α and IL-1β contents. These results are in accordance with the work of other investigators [91] and also in harmony with the results obtained by Inas et al. who demonstrated a significant increase in serum TNF-α content in indomethacin administrated shay rats [18].

Pretreatment with omeprazole significantly decreased TNF-α and IL-1β contents in comparison to the gastric ulcer control group, in accordance with studies which concluded that pretreatment with omeprazole significantly inhibited the increase in pro-inflammatory cytokine in comparison to the ulcer control group [20]. Also, in groups pre-treated with omeprazole, it was observed that there was significant reduction in the contents of TNF-α and IL-6 and at the same time significant elevation in the content of IL-10 in comparison to the indomethacin-administered rat group [113].

Misoprostol pretreatment significantly decreased TNF-α and IL-1β contents in comparison to the gastric ulcer control group.

*Althaea officinalis* pretreatment significantly decreased TNF-α and IL-1β contents in comparison to the gastric ulcer control group. Significant anti-inflammatory (acute and chronic inflammation) and anti-ulcerogenic activities were observed after administration of aqueous extract of *Althaea officinalis* flower [25]. Also, the flavonoids present in *Althaea officinalis* have anti-inflammatory and antitumor agents, moreover, some of them have antiproliferative and apoptotic effects on cancer cell lines [32].

*Solanum nigrum* pretreatment significantly decreased TNF-α and IL-1β contents in comparison to the gastric ulcer control group. *Solanum nigrum* treatment decreased the level of blood serum TNF-α, and this corresponds to triggering of apoptosis in tumor cells [88].

Heme-oxygenase-1 (HO-1) is considered to be a cytoprotective enzyme [91]. HO-1 and its metabolites have the potential to counteract the NSAIDs-induced gastric injury and show its gastro-protective effects by different mechanisms, including anti-oxidant and anti-inflammatory properties and their ability to restore mucosal blood flow [91,119].

Gaseous vasoactive mediators, such as hydrogen sulfide (H_2_S), were shown to play an important role in the mechanism of mucosal defense and gastro-protection [120,121]. H_2_S is biosynthesized by the activity of two enzymes: cystathionine-γ-lyase (CSE) and cystathionine-β-synthase (CBS) [120,121,122,123,124]. Gastric mucosa expresses both CSE and CBS, which have the ability to mediate H_2_S synthesis [121,124,125]. It has been demonstrated that H_2_S protects the GI tract against gastric damage induced by various factors [120]. H_2_S exerts a gastro-protection effect through decreased production of pro-inflammatory cytokines (IL-1β and TNF-α), reduction of gastric acid secretion (decrease acid output) along with pepsin activity, increase of endogenous prostaglandins production, regulated gastric mucosal blood flow, stimulation of bicarbonate secretion, increase of gastric juice pH and mucin concentration, decrease of reactive oxygen metabolite production, and enhancement of tissue repair by increase of GSH, CAT, and SOD enzymes activities and decrease of lipid peroxidation products [120,121,123]. In the gastrointestinal tract (GI), recent studies suggest that H_2_S may contribute to mucosal defense against injury caused by non-steroidal anti-inflammatory drugs [125]. Inhibition of H_2_S generation is a new, COX independent, mechanism of action of NSAIDs [124].

In the present work, indomethacin administration to shay rats significantly reduced the gastric mucosal HO-1 in spite of considering indomethacin as one of the cellular stress inducers. These results are in harmony with the results obtained by previous works of Allam and El-Gohary who observed that indomethacin administration reduced gastric HO-1 level significantly in comparison to the normal control group [91]. In addition, exposing rats to pyloric-ligation/indomethacin administration showed a significant decrease in CBS activity in comparison to the normal control group, in harmony with other investigations that highlighted the contribution of H_2_S to gastric mucosal defense, as well as the ability of NSAIDs to suppress endogenous H_2_S synthesis through reducing the activity and expression of CSE [122,123].

Pretreatment with omeprazole and misoprostol did not significantly affect CBS activity. But it significantly increased HO-1 activity in comparison to the gastric ulcer control group.

On other hand, our results revealed that *Althaea officinalis* and *Solanum nigrum* pretreatments significantly increased CBS and HO-1 activities in comparison to the gastric ulcer control group.

## 5. Conclusions

The present study demonstrates the in vitro and in vivo anti-oxidant potential of *Althaea officinalis* and *Solanum nigrum* extracts. In addition, it was demonstrated that oral administration of *Althaea officinalis* and *Solanum nigrum* extracts, once daily for 14 days, can protect against pyloric-ligation/indomethacin-induced gastric-ulceration in rats, probably via their anti-oxidant properties, inhibition of histamine and gastrin release, suppression of pro-inflammatory cytokines formation like TNF-α and IL-1β, and promotion of mucin, NO, PG-E_2_, and PG-I_2_ contents. Also, both extracts can increase the expressions of the protective HO-1 and CBS enzymes in gastric mucosal tissue, which is promising for further clinical trials.

## Figures and Tables

**Table 1:** Preliminary phytochemical screening of *Althaea officinalis* flower extract in hot water and *Solanum nigrum* fruit extract in 99% ethanol.

No.	Phytochemical Tests	Results	
		*Althaea officinalis*	*Solanum nigrum*
1-	Test for Carbohydrates (Molish test)	++ ve	++ ve
2-	Test for Tannins (FeCl_3_ test)	+ ve	++ ve
3-	Test for Flavonoids (NaOH/HCL test)	++ ve	++ ve
4-	Test for Saponins (Froth test)	+ ve	++ ve
5-	Test for Anthraquinon Glycosides (Born-trager test)	– ve	– ve
6-	Test for Sterols and Triterpenoids (Salkowski test)	– ve	++ ve
7-	Test for Alkaloids (Dragendorff test)	– ve	++ ve
8-	Test for Volatile Oils (Sudan III test)	+ ve	– ve
9-	Test for Cardiac Glycosides (Keller–Killiani test)	– ve	– ve
10-	Test for Cyanogenic Glycosides (Sodium picrate paper test)	– ve	– ve

Where: (++ ve) indicates a high presence of constituents, (+ ve) indicates mild presence of constituents, and (– ve) indicates absence of constituents.

**Table 2:** Scoring of histopathological examination images for stomach from different groups (pyloric ligation/indomethacin model).

Group No.	Pre Treatment	Mucosal Ulcer/Necrosis	Edema	Inflammatory Cells Infiltration
Group 1	Normal control	– ve	– ve	– ve
Group 2	Gastric ulcer control	+++ ve	+++ ve	++ ve
Group 3	Omeprazole (20 mg/kg, p.o.)	– ve	++ ve	– ve
Group 4	Misoprostol (300 µg/kg, p.o.)	– ve	++ ve	+ ve
Group 5	*Althaea officinalis* (100 mg/kg, p.o.)	– ve	+ ve	+ ve
Group 6	*Solanum nigrum* (200 mg/kg, p.o.)	– ve	++ ve	+ ve

Where: (+++ ve) indicates high presence of mucosal ulcer/necrosis, edema, or inflammatory cell infiltration, (++ ve) indicates moderate presence of mucosal ulcer/necrosis, edema or inflammatory cell infiltration, (+ ve) indicates mild presence of mucosal ulcer/necrosis, edema, or inflammatory cells infiltration, and (– ve) indicate absence of mucosal ulcer/necrosis, edema, or inflammatory cell infiltration.
